# Bio-Inspired Smart Nanoparticles in Enhanced Cancer Theranostics and Targeted Drug Delivery

**DOI:** 10.3390/jfb13040207

**Published:** 2022-10-28

**Authors:** Khushabu Gulia, Abija James, Sadanand Pandey, Kamal Dev, Deepak Kumar, Anuradha Sourirajan

**Affiliations:** 1 Faculty of Applied Sciences and Biotechnology, Shoolini University of Biotechnology and Management Sciences, Solan 173229, Himachal Pradesh, India; 2 Department of Chemistry, College of Natural Sciences, Yeungnam University, 280 Daehak-Ro, Gyeongsan 38541, Korea; 3 Department of Pharmacology & Toxicology, Wright State University, Dayton, OH 45435-0001, USA; 4 Department of Pharmaceutical Chemistry, School of Pharmaceutical Sciences, Shoolini University, Solan 173229, Himachal Pradesh, India

**Keywords:** nanotechnology, cancer theranostics, biosynthesis, therapeutics, anticancer

## Abstract

Globally, a significant portion of deaths are caused by cancer.Compared with traditional treatment, nanotechnology offers new therapeutic options for cancer due to its ability to selectively target and control drug release. Among the various routes of nanoparticle synthesis, plants have gained significant recognition. The tremendous potential of medicinal plants in anticancer treatments calls for a comprehensive review of existing studies on plant-based nanoparticles. The study examined various metallic nanoparticles obtained by green synthesis using medicinal plants. Plants contain biomolecules, secondary metabolites, and coenzymes that facilitate the reduction of metal ions into nanoparticles. These nanoparticles are believed to be potential antioxidants and cancer-fighting agents. This review aims at the futuristic intuitions of biosynthesis and applications of plant-based nanoparticles in cancer theranostics.

## 1. Introduction

In terms of mortality, cancer is one of the major causes. Despite significant advances in diagnostics and treatments, effective and safe anticancer drug delivery remains a more critical barrier in related therapies. Cancer that spreads to other body parts requires a more comprehensive and rigorous treatment regime as a first line approach, including chemotherapy either separate or combined with radiotherapy, surgeries, etc., [1]. The inability of current chemotherapies to differentiate between healthy and cancerous cells after being regularly administered is a major source of concern [2]. Standard cancer therapies sometimes fail to deliver chemotherapeutic drugs to tumor cells in an effective manner, paving the way toward new adaptations in the field of research.

Owing to fewer side effects, lower demand for heavy doses, and higher survivability, targeted therapies are preferable over traditional chemotherapy [3]. Nanotheranostics, which combine imaging and therapy, are seen to offer a viable way to solve the drawbacks of regular treatment [4].Monodispersity of size and selectivity of shape are two unique and fascinating features that make them an appealing carrier for tumor-targeted drug delivery via several modes of administration [5,6]. Nanoparticles served as a new strategy for conserving healthy cells while also eradicating cancer cells. The administration mode of nanomedicines is selected based on which method will effectively deliver nanostructures to the target part, with the maximum outcome. NPs are capable of functioning as carriers for medication and good imaging agents that bind to specific sites on cancerous tissues. Nanotheranostics is a fast-expanding field, offering a wide range of applications in the medical field, including cancer therapy [7]. Combinations of nanotechnology and pharmaceutical science can open the way for major advancements in medical research, such as effective drug delivery in cancer targeted therapy [8]. NPs can be synthesized using both physical and chemical approaches However, the chemical agents used cause environmental damage [9]. Plants (e.g., seaweeds) and microbes (e.g., bacteria) have garnered more attention than classical chemical and physical synthesis routes for metal and non-metal NPs [10].

Plant-based synthesis of nanoparticles is a way to improve the disadvantages of current procedures, thus avoiding the drawbacks of the current procedures. The combination of medicinal plants, nanoparticles, and oncology, termed “Phytonanoncology”, is providing new opportunities in cancer therapeutics. In addition to being a valuable source with anticancer potential, medicinal plants can be used to make metallic nanoparticles in an eco-friendly and green way [11]. Plant-based synthesis of nanoparticles contains biologically active components derived from natural extracts and has anticancer efficacy in cancer cells [12]. Recent studies have demonstrated that green synthesis has gained a lot of popularity since it can produce NPs with superior morphological, photochemical, photocatalytical, and electrochemical properties compared to physiochemical synthesis. This method has become one of the preferred methods for synthesizing ZnO nanoparticles [13]. Similarly, BSA (bovine serum albumin)-silver nanoparticles are potentially promising candidates for treating skin cancer in a multimodal manner [14].

This review focuses on phytocompound-based nanostructures and their synthesis, highlighting their enormous application in cancer theranostiscs and efficient drug delivery.

## 2. Phytogenic Nanoparticles in Oncology—“Phyto-Nano-Oncology”

Plants, as a natural resource with a vast diversity of phytocomponents and medicinal properties, play a crucial role in the treatment of various diseases, including cancer [15]. Certain medicinal plants produce anticancerous and tumor-fighting secondary metabolites, which further inhibit or activate various signal transduction pathways in body cells. Several phytocompounds like protein, carbohydrates, alkaloids, and organic acids act as good reducing, stabilizing, and capping agents for chloride and nitrate precursors during phytogenic synthesis of metallic nanoparticles [16]. The biogenic method allows for better control of particle size and shape compared to physical and chemical methods of nanoparticle synthesis, which are crucial for many biomedical applications. Various plant, algae, fungi, or microorganism metabolites are utilized as capping agents, reductants, and stabilizing agents for the respective NPs. Biological resources may also be used. However, they raise the issue of biosafety. In addition, maintaining cell cultures with microorganisms involves extensive and multiple steps. Thus, medicinal plants are preferable to microbes for nanoparticle synthesis [17].

**Figure 1:**
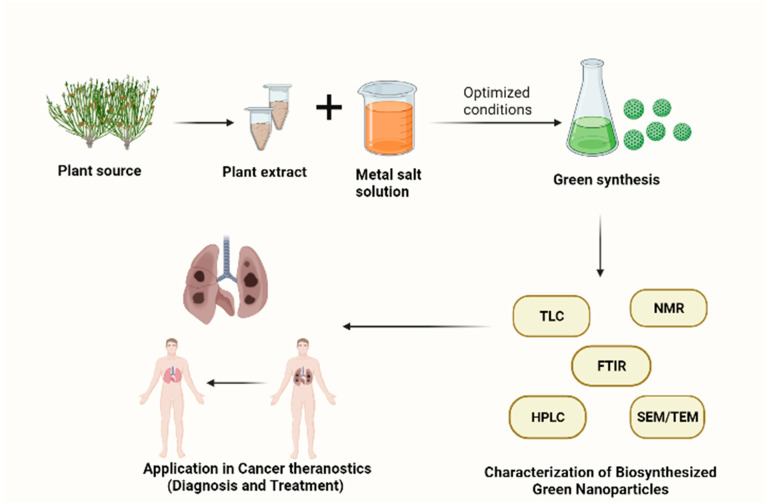
Plant-mediated process for biosynthesis of nanoparticles: optimization, characterization, and potential application in cancer theranostics. (Created with BioRender.com).

Green nanoparticle synthesis provides many additional advantages, including cultivability, cost effectiveness, stability, and rapid synthesis. Moreover, using plant compounds, different shapes and sizes of nanoparticles can be produced [10]. Metallic nanoparticles can deliver hydrophilic and hydrophobic compounds, plant-derived drugs, siRNA, peptides, antibiotics, chemotherapeutic agents, and small molecules to the targeted tumor location without causing toxicity to the healthy or surrounding tissues of the tumoral site [18]. The drug enclosed in the nanoparticles is shielded from enzyme degradation in the bloodstream. Natural extracts, essential oils, and their bioactive constituents have been shown to have multiple targeted modes of action with minimum side effects, which would be beneficial in the treatment of cancer [19]. The synthesis of plant-based nanoparticles has been shown in Figure 1.

## 3. Synthesis of Plant-Based Nanoparticles

A considerable interest has arisen in phytogenic formulation of metallic nanoparticles because the method itself is environmentally benign, simple to follow, and relies on phytochemicals like flavonoids, alkaloids, and phenol. Nanoparticle synthesis can be accomplished in two ways: top-down and bottom-up. Top-down methods seek to assemble nanoscale objects by using large microchips that are extremely controlled, while bottom-up approaches incorporate molecular-based components that are built up into more complex assemblies. Microfabrication techniques, which use externally controlled tools, are often used to cut, mill, and shape materials into the desired shape and size using the top-down approach [20,21]. Several studies have been reported in the past few years describing the relevance of MNPs such as gold (Au), silver (Ag), copper (Cu), and zinc (Zn) nanoparticles etc., because of their tiny size (nm), surface plasmon nature, and their physicochemical characteristics [22]. Nanoparticles are used across a range of industries, from cancer theranostics to drug delivery to treating wastewater. They are also used as biosensors, DNA analyzers, antibiotics, and catalysts.

Currently, plants are being used to synthesize metal nanoparticles (Table 1). As an alternative to chemical and physical methods, plants (inactivated plant tissue, plant extracts, and live plants) are increasingly being used to synthesize metal nanoparticles. Plant extracts can be used to synthesize metallic nanoparticles economically and on a large scale, so they can be used as a valuable alternative for large scale production. Nanoparticles can be synthesized using phytocompounds as both reduction and capping agents [23]. Bioreduction of metallic nanoparticles by combining different biomolecules found in plant extracts (e.g., amino acids, enzymes, vitamins, polysaccharides, proteins, and organic acids such as citrates) is chemically complex but environmentally friendly. Plants have shown great promise in accumulating and detoxifying heavy metals. Several studies have reported that plants, such as *Arabidopsis halleri* and *Thlaspi caerulescens*, detoxify and accumulate harmful metals [24]. Generally, plant-based bioreduction uses aqueous extracts to react metal salts with aqueous solutions. Because of the wide variety of chemicals involved, the process is relatively complex.One advantage of plant-assisted nanoparticle synthesis is that the kinetics of this route is far greater than that of other biosynthetic approaches that produce nanoparticles equivalent to chemical processes [25]. The nature of the plant extract, its concentration, the concentration of the metal salt, pH, temperature, and contact time are known to affect the formation of nanoparticles as well as their quantity and other characteristics.Plant parts such as fruits, leaves, stems, and roots have been widely used for green nanoparticle synthesis due to the high-quality phytochemicals they produce(Table 1). Arecoline, arecaidine, arecolidine, guvacine, guvacoline, isoguvacine, norarecaidine, and norarecoline, are some examples of alkaloids that belong to the pyridine group and play an important part in this reduction process. In the biogenesis of nanoparticles, all these bioactive compounds can cause a reduction in Au^3+^. Most plant parts, such as leaves, flowers, undergrounds (roots), and seeds, can be extracted and used as regenerative agents [26].

### Key Factors in the Synthesis of Plant-Based Nanoparticles

When optimizing phyto-NP synthesis and influencing yield and synthesis characteristics, several factors must be considered, including plant parts used for extraction, concentration of plant extract, composition of plant extract, molecular weight of biomolecules, capping agent, metal type, and the ratio of plant extracts to metal solutions.

For viable production, some external factors must also be considered, such as doping concentration, concentration of the added dopant material, pH, temperature, various solvents, light, and dissolved oxygen [47].

## 4. Drug Encapsulation in Plant-Based Nanoparticles

Encapsulation is the process of entrapping bioactive substances with a covering material in order to deliver the core at the appropriate time and location. The sizes of particles can be divided into macro (>5000 µm), micro (1–5000 µm), and nano (<1 µm) [43]. It is challenging for phytocompounds to traverse the blood-brain barrier (BBB), mucosa, gastrointestinal tract, and endothelium lining of blood vessels due to their polar nature and huge size. Additionally, they are enzymatically broken down in the digestive system. Therefore, by modifying their gastrointestinal stability, rate of dispersion, and absorption, encapsulation, or conjugation of these drugs with nanocarriers may be a different strategy to improve their bio effectiveness [48].

Generally, there are different methods used for developing micro and nanocarriers used in the encapsulation of bioactive compounds. The first category of carriers requires the use of sophisticated machinery, such as electrospinning/spraying [49], freeze-drying [50], and spray-drying [51]. Ionic gelation, an encapsulation technique carried out via electrostatic spray processes, dripping (extrusion, coextrusion) or atomization, produces nano/microcarriers [52]. Furthermore, lipid-based carriers made of fats and oils, such as liposomes and emulsions, have been successfully used to encapsulate various bio-compounds. With applications in nanomedicine, phytochemical-based nanoparticles can thus be demonstrated to be particularly effective in terms of their improved drug transport characteristics, stability, and biocompatibility [53].

## 5. Plant-Based Nanoparticles in Enhanced Cancer Imaging and Diagnosis

Detecting cancer at a late stage can make it more difficult to cure. Timely detection and diagnosis are necessary for treating cancer and preventing its complications [54]. Computed Tomography (CT), Magnetic Resonance Imaging (MRI), and Ultrasound and Positron Emission Tomography (PET) are some of the most common medical imaging procedures [55]. The lack of preciseness, effectiveness, and higher expense of the existing diagnostic methods suggest the need for novel strategies [56]. The decreased pharmacological effectiveness, expeditious removal, indefinite dissemination, and unfavorable outcomes of conventional imaging techniques suggest the necessity for a unique imaging system [57]. Biosensors are instruments used to sense signals that can be received by a detector element. The biosensors made using different nanoscale substances enhance their imaging properties to a great extent [58]. Nanoparticles such as iron, gold, titanium, silver, and copper, which are synthesized in a green manner, disperse light a million times more prominently than molecules due to their plasmon excitation, and have a pronounced ability to induce optical imaging [59].

Because of their unique characteristics, biologically synthesized silver nanoparticles would be an alternative that is more economical and straightforward, and they have proven to be highly effective with cancer theranostics [60]. Silver nanoparticles are extensively used for cancer treatment [61,62,63].The green chemistry approach is advantageous over conventional chemical methods in the synthesis of silver nanoparticles due to the following advantages: (i) simple, one-step, fast, affordable, and most reliable method; (ii) environmentally friendly due to minimal use of toxic chemicals; (iii) convenience of using bio-resources such as plants, fungi, algae, and microorganisms that act as reduction agents and stabilizing bases; and (iv) water as a universal solvent. Successful bioimaging has been demonstrated in the non-invasive in vivo imaging of silver nanoparticles using *Zinnia elegans* leaf extract in C57BL6/J mice. This can be used as a potential biosynthesized nanoparticle for future cancer imaging [64]. The intense fluorescence activity of *Olax scandens* leaf extract using methanol is already established. It was found that the silver nanoparticles made from *Olax scandens* extract incubated with A549 and B16 cells exhibited red fluorescence, and this could be used for cancer diagnosis in the future.

In physical and chemical methods of synthesis, gold nanoparticles are known to use harmful chemicals and generate an excessive amount of heat [65,66]. To alleviate these undesirable outcomes, environmentally friendly biological synthesis methods are adopted for the preparation of gold nanoparticles [67,68]. Gold nanoparticles made using plants have proven to possess properties like limited cytotoxicity, reduced immunogenicity, good stability, and permeability [69]. Using proanthocyanidin in grape seed as a reducing agent and ferric chloride as its sole precursor, iron oxide/gold combined nanoparticles were formulated. The superior superparamagnetism with remarkable CT contrast proves this green, biocompatible, and unpolluting nanoparticle synthesis as much more efficient compared to the existing imaging strategies [70]. Shiny red fluorescence was displayed in colon (COLO-205), breast (MCF7), and lung (A549) cell lines treated with green gold nanoparticles prepared using *Olax scandens*, proving their diagnostic and therapeutic activities [71]. Bifunctional imaging using CT scanning, RMI, and optoacoustic signals can be done using gold nanoparticles made utilizing different plant parts of *Hubertia ambavilla* and *Hypericum lanceolatum*. This is a rapid synthesis that does not pose any harm to our body [72]. In a recent study, gold nanoparticles made from barley leaf extract demonstrated significant biocompatibility and substantial diagnostic potential [73]. Even though current studies have proven the brilliant prospective of plant-based nanoparticles in cancer imaging, much literature explores the therapeutic potential of them. A great deal of experimentation is needed to analyze the capability of easily accessible biosensors to be used in cancer theranostics. An extensive review should be conducted to gain profound understanding of the safe, inexpensive, and biologically congruent green nanoparticles to be used as a substitute for chemically amalgamated nanoparticles in cancer diagnosis.

## 6. Targeted Drug Delivery Using Phytogenic Nanoparticles

Over 60% of anti-cancer drugs available today are derived from plants; plants have traditionally been used to treat diseases such as cancer. The discovery of anti-cancer drugs is fueled by the discovery of plants, animals, aquatic organisms, and microbes in nature [74]. Many potentially therapeutic drugs found in nature are anticancer agents, such as vinca alkaloids, taxanes and their analogs, podophyllotoxin, camptothecin (CPT) and its derivatives, anthracyclines, etc. Approximately half of the anticancer drugs approved worldwide are either natural compounds or their derivatives [75]. There are several significant disadvantages associated with most clinically used anticancer drugs, including low water solubility, incapacity for oral administration, short half-lives, and poor specificity.

**Figure 2:**
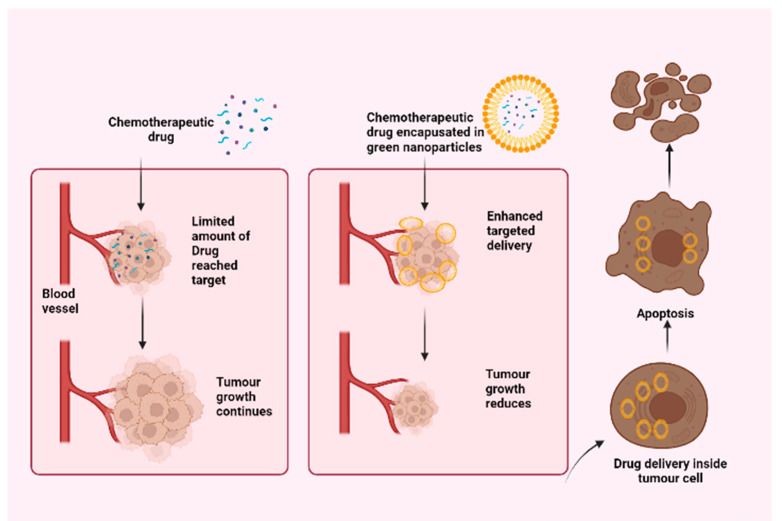
Comparison of the efficacy of chemotherapeutic drugs when incorporated into nanoparticles. (Created with BioRender.com).

The nanotech-based combinations of drugs formulated from nature-derived molecules have enormous potential for targeting tumor microenvironments in order to combat multidrug resistance (multidrug resistant) as these nanotech-based delivery systems have several advantages similar to water solubility, lower toxicity, biocompatibility, and the ability to modify their surface for further applications [76]. Drug delivery systems that are engineered at the nanoscale have been extensively studied and are by far the most advanced technology in the field of nanoparticle applications due to their potential advantages, such as the ability to modify physical properties such as solubility, release profiles, diffusion, bioavailability, and immunogenicity [77,78]. The delivery of engineering drug systems either targets a specific site or injects therapeutic agents into the site in a controlled manner [79]. Figure 2 shows the efficacy of a chemotherapeutic drug when it is encapsulated in green nanoparticles.

The use of natural compounds as a therapeutic option has gained attention in recent years. Natural compounds are being investigated for their ability to fight cancer and to soothe inflammation. Among them are curcumin, quercetin, eugenol, rosmarinic acid, and kaempferol. These phytocompounds have been encapsulated into nanoparticles to treat cancer [80]. In preclinical research for cancer treatment, biodegradable nanoparticles embedded in natural phytocompounds like resveratrol, epigallocatechin gallate, extract of pomegranate, and green tea, have been proposed.Despite natural products’ therapeutic value, their low bioavailability and solubility hinder their use. The delivery and prevention of multiple cancers may be accomplished using nanoparticles that contain naturally synthesized entities [81].As a result, nanotechnology often contributes to the success of chemopreventive interventions, and this fusion of nanotechnology and natural products is referred to as “Nano-chemo-prevention”.

SLNs (solid lipid nanoparticles) are a type of nanocarrier that contains a solid lipid matrix encapsulating natural products for anticancer therapy. The SLN system improves drug delivery systems including controlled release, stability, biocompatibility, and protection against varying drugs [82]. Another example, g-tocotrienol is a common form of vitamin E that has anticancer properties. However, its oral absorption is comparatively low, i.e., (9%). SLNs were developed as good formulations to enhance absorption into the intestine. These solid liquid nanoparticles with g-tocotrienol showed a tenfold increase in absorption as compared to the control group [83]. The nanoparticles using curcumin, silk, and chitosan were found to significantly increase curcumin uptake by MCF-7 after adding silk fibroin nanoparticles [84]. In vitro, bergamot essential oil showed increased cell death when incorporated into liposomes, which enhanced the solubility of a drug that has anticancer properties [85]. Understanding the mechanisms behind the increased efficiency of natural products, nanoparticles can be utilized to optimize delivery systems of natural phytocompounds [86]. Oncocalyxone, which isobtained from *Auxemma oncocalyx* that has anticancer properties, has also been studied using this method. Researchers have synthesized gold nanoparticles using *Punica granatum* to deliver cancer drugs to MCF-7 (breast cancer) cells [87].

**Figure 3:**
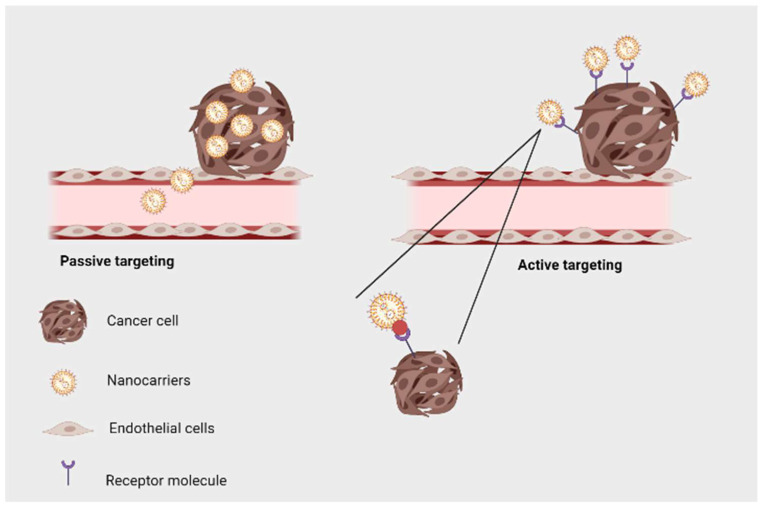
Active and passive targeting for drug delivery using nanoparticles (Created with BioRender.com).

In the drug delivery of natural phytochemicals, nanoparticles, and nanocarriers can target specific tissues or organs. This feature offers several benefits. Various targeting strategies of natural compounds can be achieved with nanoparticles. The first type of targeting involves attaching a therapeutic ligand to the surface of a nanoparticle, enhancing drug bioavailability by increasing doses that reach target tissues. Active targeting usually involves conjugating nanoparticles with proteins, peptides, antibodies, or small molecules. This enables specific tissues and organs to internalize and localize the particles. Passive targeting, on the other hand, is the delivery of drugs to a specific area of the body without the use of certain chemical interactions, but rather by relying on the intrinsic properties of the particles, such as size, shape, and surface charge. Because the drug is only being released to that specific area, the aftereffects of the drug are also reduced [88,89]. Figure 3 shows active and passive targeting of tumor cells using nanoparticles.

Additionally, as a new way, combining natural products with radiotherapy, chemotherapy, and immunotherapy can enhance the synergistic effect of natural products, restricting the doses for patients and reducing toxicity [90].

## 7. Insights on Different Cancer Pathways Targeted by Plant-Based Nanoparticles

Plant-based nanoparticles can be modified to perform specific functions in target tissues, such as stimulating stem cell proliferation, reducing colitis injury, activating intrinsic and extrinsic apoptosis pathways, and inhibiting tumor growth. When internalized, they can induce stem cell proliferation, reduce colitis injury, and stimulate apoptosis pathways.

It has been shown that AgNPs interact with the cellular materials of the cell, destruct DNA, and cause cell death. Alternatively, they can disrupt ATP synthesis, cause mitochondrial respiratory disturbances and damage DNA [91]. As reported in another study, AgNPs can enter cancerous cells and interfere with RNA topoisomerase enzyme activity and gene transcription through mutual contact. The destruction of cancerous cells is therefore more delicate than that of normal cells [92]. *Houttuynia cordata* (Hc-CuONPs) plant extract was used in another study to synthesize copper nanocomplexes that promote apoptosis by targeting the PI3K/Akt signaling pathways in HeLa cells [93]. Nanoparticles loaded with curcumin can cross the blood-brain barrier, allowing them to reach the brain tissue and remain there for longer than curcumin alone. Several studies have demonstrated the anti-glioma efficacy of curcumin-loaded nanocarriers. NP-encapsulated curcumin affects CD133+ stem cell populations [94]. As compared to the equivalent dose of free resveratrol, methoxypoly(ethylene glycol)-polycaprolactone (mPEG-PCL) NPs encapsulating resveratrol induce a higher level of apoptosis in glioma cells, resulting in greater ROS release [95].

## 8. Limitations of Plant-Based Metallic Nanoparticles

Nanoparticle research and prospective uses have expanded dramatically in recent years. Metallic nanoparticles have been synthesized using a variety of biological sources, including plants, bacteria, fungus, and yeast. Despite its widespread manufacturing and subsequent uses, a few obstacles remain, such as adjusting the process parameters (temperature, pH, etc.) and reactants necessary for NP characterization. Furthermore, green synthesis methods are not nearly as cost-effective or comparable to traditional methods for large-scale nanoparticle manufacturing. Another crucial issue that must be investigated is the separation and purification of NPs from the reaction mixture [96]. Although the green synthesis technique has received substantial attention from researchers, and a broad range of plant extract-based NPs have been synthesized and reviewed so far, this study topic still needs to be investigated in order to synthesize more efficient theranostics.

## 9. Conclusions and Future Perspectives

The field of nanoscience has brought significant advancements in medicine, particularly for cancer. In recent years, scientists have been focusing on the technology of green synthesis to synthesize nanoparticles by adopting an eco-friendly approach. In addition to being more stable when compared to those produced by other organisms, plant extracts can reduce metal ions faster than fungi or bacteria. Therefore, plant extracts are highly recommended for scale-up and industrial production of well-dispersed metal nanoparticles. In addition to the diagnosis and treatment of diseases, phyto-based nanotechnology can be used to deliver drugs and to analyze biomedical images. The advancement of nanotechnology will offer a better chance to adopt suitable therapeutic strategies while simultaneously targeting several molecules of cancer samples. The green route is expected to lead to exponential applications of nanoparticles, but there is a need to be concerned about the long-term effects of these on animals and humans as well as the accumulation of these in the environment. A large-scale, successful clinical trial is necessary in order to develop plant-based nanoparticles that can be used in personalized cancer therapies.

## Figures and Tables

**Table 1:** List of previous studies utilizing plant active compounds for the biosynthesis of nanoparticles as cancer therapeutics and diagnosis.

Plant Name	Part Used	Type of Nanoparticles	Cancer Cell Lines	References
*Benincasa hispida*	Fresh peel	Gold (Au)	HeLa cells and normal osteoblast cell lines	[27]
*Butea monosperma*	Leaves	Gold (Au)	Cancer cell lines (B16F10, MCF-7, HNGC2, and A549)	[28]
*Ocimum sanctum*	Leaves	Gold (Au)	Dalton’s lymphoma	[29]
*Bauhinia tomentosa Linn*	Leaves	Gold (Au)	A549, HEp-2, and MCF-7 cells	[30]
*Cassia tora*	Leaves	Gold (Au)	Colon cancer cells	[31]
*Hibiscus sabdariffa*	Leaves	Gold (Au)	U87 cell line	[32]
*Moringa oleifera*	Leaves	Gold (Au)	A549 and SNO cells	[33]
*Piper longum*	Fruit	Silver (Ag)	MCF-7	[34]
*Plumeria alba*	Flower	Silver (Ag)	COLO-205	[35]
*Rosa indica*	Petal	Silver (Ag)	HCT 15	[36]
*Sesbania grandifloria*	Leaves	Silver (Ag)	MCF-7	[37]
*Rheum emodi*	Root	Silver (Ag)	MCF-7	[38]
*Solanum trilobatum*	Fruit	Silver (Ag)	MCF-7	[39]
*Quercus*	Fruit	Silver (Ag)	MCF-7	[40]
*Saccharum officinarum*	Juice	Zinc (Zn)	MCF-7	[41]
*Cannabis sativa*	Leaves	Zinc (Zn)	A549	[42]
*Catharanthus roseus*	Leaves	Zinc (Zn)	MCF-7	[43]
*Calotropis gigantea*	Leaves	Zinc (Zn)	A549	[44]
*Saraca asoca*	Flowers	Zinc (Zn)	WEHI-3 cells	[45]
*Withania somnifera*	Leaves	Zinc (Zn)	WEHI-3 cells	[46]
